# Habitual Sleep Patterns and Chronic Sleep Problems in Relation to Sex, Age, and Circadian Preference in a Population-Based Sample of Norwegian Adults

**DOI:** 10.3390/clockssleep5010003

**Published:** 2023-01-06

**Authors:** Ingvild West Saxvig, Bjørn Bjorvatn, Siri Waage

**Affiliations:** 1 Norwegian Competence Center for Sleep Disorders, Haukeland University Hospital, 5021 Bergen, Norway; 2 Department of Global Public Health and Primary Care, University of Bergen, 5020 Bergen, Norway; 3 Department of Psychosocial Science, University of Bergen, 5020 Bergen, Norway

**Keywords:** sleep, sleep patterns, sleep duration, social jetlag, sleep problems, circadian preference, sex, age

## Abstract

Sleep patterns and problems vary in relation to internal (e.g., sex, age, circadian preference) and external (e.g., social structures) factors. The main aim of the present study was to describe habitual sleep patterns and chronic sleep problems in a population-based sample of Norwegian adults. During spring 2022, a sample of 1028 adults completed an online survey on sleep habits and problems. Response rate was 33.5%. The survey included the Munich ChronoType Questionnaire and items on circadian preference and chronic sleep problems. Mean workday sleep duration was 7:19 h (±199 min), and shorter in males (*p* = 0.035) and evening persons (*p* = 0.003). Short workday sleep duration (<6 h) was reported by 3.1% and was associated with evening preference (*p* = 0.001). Mean social jetlag was 0:51 h (±75 min), and longer in males (*p* = 0.036), younger adults (*p* < 0.001) and evening persons (*p* < 0.001). Long social jetlag (≥2 h) was reported by 11.2% and associated with younger age (*p* < 0.001) and evening preference (*p* < 0.001). Chronic sleep problems (≥3 months) were reported by 44.1%, and associated with female sex (*p* < 0.001) and evening preference (*p* = 0.002). Results underscore the importance of considering evening circadian preference as a risk factor for short workday sleep duration, long social jetlag and self-reported chronic sleep problems.

## 1. Introduction

Adequate sleep is crucial for health and daytime functioning. What comprises adequate sleep, however, varies in relation to inter- and intra-individual differences. With respect to age, the US National Sleep Foundation recommends that adults obtain at least 7 h of sleep each night, but adds that 6+ hours may be appropriate in younger and middle-aged adults (18–64 years) and 5+ hours in older adults (65 years or older) [1]. Short sleep duration, in particular sleep durations < 6 h, is associated with a wide range of negative outcomes in terms of neurobehavioral performance, metabolism, cardiovascular health, mortality and psychological health [2]. Still, epidemiological studies from different countries, including Norway, have indicated that many adults sleep less than what is currently recommended [3,4,5,6,7].

Circadian preference reflects the preferred timing for sleep and wake, and changes gradually from an evening preference in younger adults towards a morning preference in the elderly [8]. An evening circadian preference is associated with short workday sleep duration (late bed times combined with early rise times) and social jetlag (difference between internal and social timing for sleep, measured by the free day/workday discrepancy in sleep timing) [9]. Thus, although sleep duration generally appears to decline from early adulthood to older age [1,10], many studies have found shorter workday, but not free day, sleep durations in adolescents and young adults compared to older adults [11,12]. A long social jetlag, in particular longer than 2 h, appears to be associated with adverse health measures [13,14,15]. There also appears to be sex-related differences in sleep and circadian preference. Several studies find that women more often report a morning- and men more often an evening circadian preference [8,16,17], as well as longer sleep duration in females than in males [4,18,19]. However, other studies have failed to demonstrate such differences [20,21,22], or found that the differences disappear following menopause in women [8,16,23,24,25]. Studies more univocally show that women report poorer sleep quality and more sleep problems than men [4,22,26,27].

The prevalence of sleep problems appears to have increased during the last decades [4,5,28,29,30,31]. Such secular trends in sleep problems are believed to reflect the dark side of the modern 24 h society (e.g., busy schedules, shift work, entertainment available at all hours and altered patterns of light exposure), which may affect both the time we allocate for sleep, our ability to sleep, and the circadian regulation of sleep. In line with such notions, some studies have also found a decline in sleep duration in the adult population over time [5,6,32]. Findings are, however, somewhat mixed [33], and a systematic review concludes that sleep duration has been relatively stable over time [34]. More recently though, population-based studies have indicated changes in sleep in relation to the COVID-19 pandemic and associated social restrictions [35,36], particularly among those with an evening circadian preference [37].

Given the unresolved issues regarding inter- and intra-individual differences in sleep, it seems important to accumulate population-based data on sleep patterns and sleep problems, stratified by age, sex and circadian preference. Furthermore, contemporary, population-based data on sleep can contribute to shed light on secular trends in sleep, not least considering the recent COVID-19 pandemic and its possible effects on sleep. In the present study, we therefore aimed to (1) describe the distribution of circadian preference in a population based sample, stratified by sex and age, (2) describe habitual sleep patterns (timing, regularity, latency and duration), stratified by sex, age and circadian preference and, (3) explore the prevalence of self-reported chronic sleep problems (duration ≥ 3 months), short workday sleep duration (<6 h) and long social jetlag (≥2 h) in relation to sex, age and circadian preference.

## 2. Materials and Methods

During spring 2022, a population-based sample of Norwegian adults were invited to participate in a web-based survey on sleep and health. The survey was technically administered by the opinion-research institute Kantar TNS, Oslo, Norway. Targeting 1000 responders representative of the Norwegian population, the sample was randomly selected by sex, age and geography (stratified random sampling) from Kantar’s web panel, which is a consent-based database containing personal information of about 40,000 participants recruited to reflect the demography of the Norwegian population. The sole inclusion criterion was age ≥ 18 years. There were no exclusion criteria. When collecting the data, demographic groups with poor response rates were oversampled in order to obtain a sample representative for the population, and a weight factor was calculated, controlling for age, sex and county of residence. Out of 3071 invited participants, a total of 1028 responded to the study, yielding a response rate of 33.5%.

### 2.1. Instruments

#### 2.1.1. Sociodemographic Variable

Relevant sociodemographic parameters, including sex, age, county of residence and educational level were collected. For the purpose of analyses, age was grouped into the following categories: 18–35 years, 36–50 years, 51–65 years, 66+ years. Educational level was grouped into the following categories: Primary school, secondary school, college/university.

#### 2.1.2. Circadian Preference

Circadian preference was addressed with the single item “Are you a morning- or an evening person?”, with the following response options: “I am very alert and active in the morning and sleepy early in the evening (definitely morning preference); I am rather alert in the morning and rather sleepy in the evening (morning preference); Neither morning- nor evening person (intermediate preference); I am rather alert in the evening and rather sleepy in the morning (evening preference); I am very alert and active in the evening and sleepy in the morning (definitely evening preference)”.

#### 2.1.3. Habitual Sleep Patterns

The questionnaire included items on sleep habits, adapted from the Munich ChronoType Questionnaire (MCTQ) [35], asking about habitual sleep for workdays and free days, respectively. The basic items used were shuteye time (ST, response options at 15 min intervals), sleep onset latency (SOL, response options at 5 min intervals from 0 min to 5 h) and wake up time (WUT, response options at 15 min intervals). Based on these variables, we calculated sleep onset (SO), sleep duration (total sleep time, TST) and midsleep (MS) for workdays and free days, respectively, as well as the Δ values (free day—workday) for the sleep timing parameters, out of which ΔMS reflects the social jetlag and ΔSO the sleep corrected social jetlag [36]. See Table 1 for a detailed description of the reported and computed sleep habit parameters. Parameters on habitual sleep pattern reported in the present study include parameters on sleep timing (SO, MS, WUT, on workdays and free days, respectively), sleep regularity (ΔSO, ΔMS, ΔWUT), sleep onset latency (SOL on workdays and free days, respectively) and sleep duration (TST on workdays and free days, respectively). In addition, we identified responders with short workday sleep duration (<6 h, based on recommendations by US National Sleep Foundation [1]) and long social jetlag (≥2 h, based on previous studies and suggestions [13,14,15]).

#### 2.1.4. Chronic Sleep Problems

To address chronic sleep problems, the questionnaire included the following items: “Do you currently experience sleep problems?” (response options: No; Yes, a little; Yes, some; Yes, much; Yes, very much; I don’t know), and for those responding Yes (a little, some, much or very much) a follow-up question was added: “For how long have you experienced sleep problems” (response options: Less than 3 months; 3 months up to 1 year; 1 year up to 5 years; 5 years or more). Those who reported sleep problems with a duration of 3 months or longer, were classified as having chronic sleep problems.

### 2.2. Statistics

IBM SPSS Statistics 26 (SPSS Inc., Chicago, IL, USA) was used for statistical analyses. Analyses were performed using a weight factor calculated in accordance with the population distribution of age, sex and county of residence, in order to correct for potential divergence between the sample and the demography of the general population. Circadian preference was explored in relation to sex and age, using chi-square analyses. Habitual sleep patterns were explored in relation to sex, age and circadian preference using *t*-tests (sex) and 1-way ANOVAs (age and circadian preference). To account for multiple comparisons of means (13 parameters × 3 independent variables = 39 tests), *p*-values from these analyses were adjusted using False Discovery Rate (FDR), calculated as *p*-value × 39 tests/[rank] [37]. Chronic sleep problems (duration ≥ 3 months), short workday sleep duration (<6 h) and long social jetlag (≥2 h) were explored in relation to age, sex and circadian preference using chi-square analyses. In addition, we conducted logistic regression analyses with chronic sleep problems, short workday sleep duration and long social jetlag as dependent variables, and sex, age, education and circadian preference as independent variables. The regression analyses were conducted both crude (independent variables entered separately) and adjusted (independent variables entered simultaneously). Significance level was set to 0.05 for all analyses.

## 3. Results

The sample comprised 49.6% females, had a mean age of 48.6 years ± 16.9 (range 18–89 years), and 58.4% had college or university education. Sociodemographic (sex, age, and education) and clinical (circadian preference, chronic sleep problems, short workday sleep duration, long social jetlag) characteristics of the sample are displayed in Table 2.

### 3.1. Circadian Preference

A total of 37.1% reported a morning preference (either definitely morning or morning preference), 27.9% reported an intermediate preference (neither morning nor evening preference), and 35.0% reported an evening preference (either definitely evening or evening preference), see Table 2 for further details. There were no sex-related differences in circadian preference, neither overall in the sample (χ^2^ = 5.881, *p* = 0.208) nor within either of the age groups 18–35 years (χ^2^ = 8.886, *p* = 0.064), 36–50 years (χ^2^ = 3.623, *p* = 0.459), 51–65 years (χ^2^ = 7.174, *p* = 0.127) or 66+ years (χ^2^ = 3.859, *p* = 0.425). There was, however, an association between circadian preference and age (χ^2^ = 55.614, *p* < 0.001). The two younger age groups (18–35 and 36–50 years) more often reported an evening/definitely evening preference (43.3% and 42.5% in the two age groups, respectively) than a morning/definitely morning preference (31.1% and 30.3% in the two age groups, respectively), whereas the older age groups (51–65 and 66+ years) more often reported a morning/definitely morning preference (45.3% and 41.9% in the two age groups, respectively) than an evening/definitely evening preference (30.9% and 20.1% in the two age groups, respectively).

### 3.2. Habitual Sleep Patterns

Habitual sleep patterns, overall and in relation to sex, age and circadian preference, are displayed in Table 3. Summarized, females had earlier sleep onset on free days, longer sleep duration on workdays and shorter social jetlag compared to males, the younger age groups had later sleep timing and longer sleep duration on free days, and less regular sleep compared to the older age groups, and those with later circadian preference had later sleep timing and longer sleep onset latency both on workdays and free days, shorter sleep duration on workdays and less regular sleep compared to those with earlier circadian preferences.

### 3.3. Chronic Sleep Problems

Altogether, 44.1% reported chronic sleep problems (duration ≥ 3 months). The prevalence of chronic sleep problems differed in relation to sex (*p* < 0.001), age (*p* = 0.010) and circadian preference (*p* = 0.002), see Table 4 for details. In the adjusted logistic regression analyses, chronic sleep problems were associated with female sex (OR 1.80) and a definitely evening preference (OR 1.68, intermediate preference as reference), see Table 5 for details.

### 3.4. Short Workday Sleep Duration

Only 3.1% of the sample reported short sleep duration on workdays (<6 h). The proportion did not differ in relation to sex (*p* = 0.399) or age (*p* = 0.728), but it differed in relation to circadian preference (*p* = 0.001), see Table 4 for details. In the adjusted logistic regression analyses, short sleep duration on workdays was associated with a definitely evening preference (OR 3.56, intermediate preference as reference), see Table 5 for details.

### 3.5. Long Social Jetlag

A total of 11.2% reported long social jetlag (≥2 h). The proportion did not differ in relation to sex (*p* = 0.526), but it differed in relation to age (*p* < 0.001) and circadian preference (*p* < 0.001), see Table 4 for details. In the adjusted logistic regression analyses, long social jetlag was less common in participants with higher age (OR 0.59 in adults 36–50 years, OR 0.40 in adults 51–65 years and OR 0.03 in adults 66+ years, adults 18–35 as reference), and less common in participants with a morning preference and more common in participants with an evening preference (OR 0.17 in definitely morning persons and OR 3.94 in definitely evening persons, intermediate persons as reference), see Table 5 for details.

## 4. Discussion

This population-based study found that circadian preference was distributed quite evenly amongst Norwegian adults, with ~37% reporting being morning, ~28% intermediate and ~35% evening persons, but with a skew towards an evening preference in the younger and towards a morning preference in the older age groups. Mean workday sleep duration was 7:19 h, and shorter in males compared to females and in later compared to earlier circadian preferences. Short workday sleep duration (<6 h) was reported by only ~3%, and was most common in definitely evening persons. Mean social jetlag was shorter than one hour (0:51 h), and longer in males, younger adults and those with a later circadian preference. Long social jetlag (≥2 h) was reported by ~11%, and was most common in definitely evening persons and least common in definitely morning persons and the older age groups. Chronic sleep problems (duration ≥ 3 months) were reported by ~44%, and were most common in females and definitely evening persons. Providing contemporary population-based data on sleep patterns and sleep problems, results from the present study add importantly to a field where several unresolved issues remain with respect to inter- and intra-individual differences in sleep habits as well as possible secular trends in sleep. In particular, stratification by circadian preference represents a novel approach in sleep habits epidemiology, and underscores the importance of considering evening circadian preference as a risk factor for short workday sleep duration, long social jetlag and self-reported chronic sleep problems.

The finding that younger adults more often reported having an evening- and older adults having a morning preference is in line with previous studies [23]. Interestingly however, data from the present study show a similar distribution of circadian preference in the 18–36 year olds and the 36–50 year olds, which may indicate that the gradual shift from eveningness in adolescents towards morningness in the elderly, accelerates in middle aged, as also noted by Adan and colleagues in their review on circadian typology [8]. The lack of association between circadian preference and sex is in line with findings by Paine and colleagues [21], but contrasts previous studies reporting more morningness in females [16], more morningness in pre-menopausal females [23,25] or even more eveningness in females [40]. Thus, whereas a relationship between age and circadian preference seems largely supported by the present study, the possible presence and nature of a relationship between sex and circadian preference seems more unclear and warrants further investigation in future population-based studies.

As expected, stratification of habitual sleep patterns by circadian preference revealed later sleep timing, less sleep regularity, longer sleep latency and shorter workday sleep duration in those with later circadian preferences. Individuals with a late circadian preference are often unable to compensate for early workday rise times by early sleep onset, resulting in curtailed workday sleep duration and long social jetlag [9]. This explanation is supported by the finding that sleep latency was longer with later circadian preference, even despite later bedtimes. There were also clear associations between habitual sleep patterns and age. Notably, sleep timing on free days was earlier and social jetlag shorter with increasing age. These findings are in concordance with the reported age-related differences in circadian preference. Still, the odds ratio for a long social jetlag remained lower with older age also after controlling for circadian preference, sex and education (adjusted OR 0.03 for adults 66+ years). Since social jetlag reflects the combined effects of circadian preference and social obligations, earlier and more regular sleep patterns in older adults can be explained by differences in social expectations and obligations with age. Similarly, younger adults had longer sleep duration than older adults on free days, but not workdays. These findings support previous research showing that sleep duration decrease with age [10]. It is not obvious whether the age-related differences in free day sleep duration reflect differences in sleep need or sleep ability [41]. It is possible that younger adults need more sleep than older, and thus suffer sleep curtailment on workdays with subsequent sleep rebound on free days, despite similar workday sleep durations across the age groups. However, it is also possible that younger adults have larger ability to extend sleep beyond their actual need than older adults. We recommend that future studies include items on individual perceived sleep need, in order to better identify those with sub-optimal sleep durations. Stratification of habitual sleep by sex showed that males and females had quite similar sleep patterns, but with slightly longer workday sleep duration in females than males. Longer sleep duration in females has been reported in several studies [4,18,19], and it is not clear whether this difference reflects differences in sleep need or sleep behaviour. The proportions reporting short sleep duration or long social jetlag did not differ between the sexes.

Despite the finding of only minor differences in sleep patterns between the sexes, chronic sleep problems were much more common among females, which is in concordance with a large number of studies [4,22,26,27]. Both biological (e.g., hormones) and psychosocial (e.g., social obligations) factors may contribute to these differences, in addition to sex-related differences in mental and somatic health problems and dissimilarities in how men and women report their symptoms and problems. In line with previous studies, chronic sleep problems were also strongly associated with circadian preference, and most often reported by definitely evening types [40]. Self-reported sleep problems among evening types may reflect their characteristic sleep pattern, with long sleep latency, curtailed workday sleep and long social jetlag. It can also reflect certain sleep disorders. Extreme eveningness may predispose for delayed sleep-wake phase disorder (DSWPD) [42], and possibly contribute to conditioning of insomnia, due to frequent experiences of being unable to fall asleep in due time [43,44]. Furthermore, previous studies have shown that eveningness is related to certain personality traits and mental health issues, which may directly or indirectly have consequences for sleep and sleep problems [8].

Previous studies have indicated a slight decrease in adults’ sleep duration during the last decades [5,6,32]. Findings have however, not been univocal, and in a systematic review from 2012, Bin and colleagues concluded that sleep duration has in fact remained relatively stable since the 1960s [34]. In the present study, mean sleep duration was slightly longer than what has previously been reported amongst Norwegian adults [4,18]. This discrepancy is likely related to the age ranges in the respective samples, in that only the present study included participants younger than 40 years. In fact, for the older age groups, sleep durations in the present study are relatively in concordance with previous findings [4,18]. Regarding secular trends in sleep problems, the magnitude of different assessment methods that has been used to explore this issue complicates comparisons between studies. Still, the finding that more than two out of five adults in the present study reported experiencing sleep problems for 3 months or longer is worrying, and adds to previous Norwegian studies showing that a large proportion of adults experience sleep problems [4,28,31]. Chronic sleep problems represent a major risk factor for poor health and daytime functioning, thus comprising a major public health concern.

The study has several methodological strengths. First, sleep patterns were described in relation to circadian preference, a trait that is often overlooked in epidemiological studies on sleep in adult populations, but that may have huge impact on sleep. Second, habitual sleep patterns were measured using separate items for workdays and free days, allowing a more detailed assessment of sleep patterns and sleep regularity, including social jetlag. Another strength of the study, comprise the relatively large sample, drawn and weighted to reflect the demography of the adult Norwegian population. The study also has several limitations. First, it is important to note that the present study was based on self-report. Thus, neither sleep habits, circadian rhythm nor sleep problems have been corroborated by objective data. Items on sleep habits were adapted from the Norwegian version of the MCTQ, which has not been formally validated. Circadian preference was addressed with a single item, rather than with a validated questionnaire. Still, morning and evening preferences were relatively evenly distributed in the sample, which is in line with previous reports [8]. Chronic sleep problems were addressed with only two items, and not corroborated by validated questionnaires, clinical interviews or objective measures of sleep. Thus, we were not able to identify the cause of these subjective sleep problems (e.g., sleep disorders such as insomnia, sleep apnea or circadian rhythm sleep-wake disorders). Nor did we include questions on general mental or somatic health, or any use of medication (i.e., hypnotics). Thus, the participants reporting chronic sleep problems likely comprised a heterogeneous group with diverse aetiologies, limiting our ability to draw conclusions with respect to specific sleep disturbances. Moreover, since no item on wakefulness during the sleep period was included, sleep duration is likely overestimated, particularly for those with chronic sleep problems, which may have long or frequent awakenings from sleep. The survey did not include items on several other sociodemographic or lifestyle parameters that may influence sleep and circadian rhythm, such as consumption of caffeine/nicotine/alcohol, physical activity, media use before bedtime, work schedules, ethnicity, cultural background, etc. Furthermore, although a relatively large sample, statistical power was reduced for the subgroup analyses, thus the risk for false negatives should be kept in mind when interpreting the data. The common method bias inherent to cross sectional designs should also be kept in mind.

## 5. Conclusions

The current study provides novel data on habitual sleep patterns and chronic sleep problems in relation to sex, age and circadian preference in a representative sample of the Norwegian adult population. Results showed that sleep patterns and problems varied in relation to sex and age, and in particular in relation to circadian preference. These results underscore the importance of considering evening circadian preference as a risk factor for short workday sleep duration, long social jetlag and self-reported chronic sleep problems.

## Figures and Tables

**Table 1:** Summary of the sleep habit parameters addressed in the survey.

Parameter	Abbreviation	Statement	Computation
Shuteye time ^1^	ST	At what time do you usually attempt to go to sleep?	-
Sleep onset latency ^1^	SOL	How long does it usually take you to fall asleep?	-
Wake up time ^1^	WUT	At what time do you usually wake up?	-
Sleep onset ^1^	SO	-	ST + SOL
Sleep duration ^1^	TST	-	WUT − SO
Midsleep ^1^	MS	-	SO + TST/2
Sleep corrected social jetlag	ΔSO	-	SO_f_ − SO_w_
Social jetlag	ΔMS	-	MS_f_ − MS_w_
-	ΔWUT	-	WUT_f_ − WUT_w_

^1^ Reported/computed for workdays (_w_) and free days (_f_), respectively.

**Table 2:** Sociodemographic and clinical characteristics of the sample (*n* = 1028). Data are presented both in its crude form and weighted for age, sex and county of residence.

		Crude	Weighted
Sex			
	Female	509 (49.5%)	510 (49.6%)
	Male	519 (50.5%)	518 (50.4%)
Age			
	18–35 years	195 (19.0%)	285 (28.7%)
	36–50 years	276 (26.8%)	247 (24.0%)
	51–65 years	370 (36.0%)	296 (28.8%)
	66+ years	187 (18.2%)	200 (19.4%)
Education			
	Primary school	42 (4.1%)	46 (4.5%)
	Secondary school	378 (36.8%)	381 (37.1%)
	College/university	608 (59.1%)	600 (58.4%)
Circadian preference (*n* = 1018)			
	Definitely morning	116 (11.4%)	110 (10.8%)
	Morning	270 (26.5%)	268 (26.3%)
	Intermediate	279 (27.4%)	284 (27.9%)
	Evening	223 (21.9%)	225 (22.1%)
	Definitely evening	132 (12.9%)	132 (12.9%)
Chronic sleep problems (≥3 months)			
	No	567 (55.2%)	575 (55.9%)
	Yes	461 (44.8%)	453 (44.1%)
Short workday sleep duration (<6 h)			
	No	956 (96.8%)	955 (96.9%)
	Yes	32 (3.2%)	30 (3.1%)
Long social jetlag (≥2 h)			
	No	870 (89.7%)	858 (88.8%)
	Yes	100 (10.3%)	109 (11.2%)

**Table 3:** Habitual sleep patterns (timing, regularity, latency, duration) in a population-based sample of Norwegian adults, overall and in relation to sex, age and circadian preference.

		Sleep Onset (SO)			Midsleep (MS)			Wake Up Time (WUT)			Sleep Onset Latency (SOL)		Sleep Duration (TST)	
		Workday	Free Day	ΔSO	Workday	Free Day	ΔMS	Workday	Free Day	ΔWUT	Workday	Free Day	Workday	Free Day
Total		23:44 ± 86	0:17 ± 86	0:34 ± 56	3:23 ± 89	4:15 ± 84	0:51 ± 75	7:04 ± 124	8:12 ± 101	1:08 ± 119	0:22 ± 31	0:20 ± 29	7:19 ± 119	7:53 ± 86
Sex														
	Female	23:39 ± 85	0:09 ± 74	0:30 ± 62	3:23 ± 92	4:09 ± 73	0:46 ± 81	7:08 ± 136	8:10 ± 97	1:01 ± 128	0:23 ± 30	0:21 ± 24	7:28 ± 163	7:59 ± 92
	Male	23:49 ± 88	0:26 ± 96	0:37 ± 49	3:24 ± 86	4:20 ± 93	0:57 ± 69	6:59 ± 110	8:15 ± 105	1:16 ± 109	0:20 ± 33	0:19 ± 32	7:10 ± 100	7:48 ± 79
	*p* (*t*-test)	0.127	**0.005**	0.075	00.914	0.066	**0.036**	0.317	0.506	0.067	0.225	0.502	**0.035**	0.090
	*n*	994	1000	983	985	982	966	1009	1002	993	1007	1006	985	982
Age														
	18–35	23:39 ± 77	0:24 ± 87	0:46 ± 50	3:27 ± 77	4:40 ± 85	1:14 ± 58	7:17 ± 94	8:58 ± 98	1:41 ± 85	0:22 ± 26	0:18 ± 14	7:36 ± 73	8:31 ± 72
	36–50	23:46 ± 106	0:25 ± 101	0:39 ± 54	3:22 ± 106	4:25 ± 102	1:04 ± 71	6:58 ± 138	8:25 ± 122	1:28 ± 110	0:21 ± 22	0:18 ± 18	7:11 ± 127	7:59 ± 95
	51–65	23:44 ± 91	0:14 ± 83	0:30 ± 70	3:20 ± 102	4:02 ± 72	0:41 ± 101	6:55 ± 159	7:48 ± 80	0:53 ± 167	0:23 ± 37	0:21 ± 33	7:12 ± 162	7:34 ± 77
	66+	23:49 ± 60	0:02 ± 66	0:15 ± 32	3:27 ± 50	3:43 ± 53	0:16 ± 23	7:04 ± 64	7:23 ± 67	0:17 ± 30	0:21 ± 39	0:24 ± 44	7:16 ± 75	7:18 ± 83
	*p* (ANOVA)	0.681	**0.036**	**<0.001**	0.808	**<0.001**	**<0.001**	0.197	**<0.001**	**<0.001**	00.920	0.174	0.078	**<0.001**
	*n*	994	999	983	985	982	967	1009	1002	993	1007	1006	985	982
Circadian preference														
	Def. morning	22:53 ± 63	23:20 ± 66	0:27 ± 36	2:39 ± 71	3:15 ± 76	0:36 ± 35	6:25 ± 115	7:10 ± 121	0:45 ± 49	0:17 ± 30	0:17 ± 30	7:32 ± 120	7:49 ± 125
	Morning	23:14 ± 52	23:42 ± 54	0:29 ± 33	2:57 ± 67	3:40 ± 47	0:43 ± 65	6:40 ± 117	7:37 ± 61	0:55 ± 125	0:18 ± 27	0:16 ± 22	7:28 ± 125	7:55 ± 67
	Intermediate	23:48 ± 100	0:16 ± 93	0:27 ± 69	3:32 ± 110	4:12 ± 89	0:39 ± 93	7:18 ± 160	8:09 ± 109	0:51 ± 148	0:23 ± 28	0:22 ± 31	7:29 ± 151	7:52 ± 91
	Evening	23:56 ± 62	0:39 ± 64	0:43 ± 48	3:33 ± 59	4:39 ± 64	1:06 ± 56	7:10 ± 71	8:37 ± 80	1:28 ± 81	0:23 ± 32	0:20 ± 25	7:13 ± 65	7:58 ± 70
	Def. evening	0:58 ± 107	1:47 ± 96	0:49 ± 82	4:19 ± 98	5:42 ± 85	1:22 ± 93	7:42 ± 109	9:39 ± 97	1:57 ± 116	0:31 ± 44	0:26 ± 40	6:40 ± 92	7:48 ± 95
	*p* (ANOVA)	**<0.001**	**<0.001**	**0.001**	**<0.001**	**<0.001**	**<0.001**	**<0.001**	**<0.001**	**<0.001**	**0.003**	**0.030**	**0.003**	0.867
	*n*	988	993	977	979	977	962	1003	997	988	1001	999	979	977

All parameters are presented for workdays and free days, separately. Regularity in sleep timing is presented as a Δvalue (free days—workdays). Note that midsleep (MS) on free days reflects the chronotype, ΔMS reflects the social jetlag and ΔSO reflects the sleep corrected social jetlag (SJL_sc_) [38]. All *p*-values are FDR adjusted for 39 tests [39]. Significant *p*-values in bold.

**Table 4:** Prevalence of self-reported chronic sleep problems (duration ≥ 3 months), short workday sleep duration (< 6 h) and long social jetlag (≥ 2 h) in relation to sex, age and circadian preference.

		Chronic Sleep Problems			Short Workday Sleep Duration			Long Social Jetlag		
		No	Yes	Chi-Square	No	Yes	Chi-Square	No	Yes	Chi-Square
Sex										
	Female	252 (49.4%)	258 (50.6%)	***p*** **< 0.001**	466 (96.5%)	17 (3.5%)	*p* = 0.399	425 (89.5%)	50 (10.5%)	*p* = 0.526
	Male	323 (62.2%)	196 (37.8%)	χ^2^ = 17.157	488 (97.4%)	13 (2.6%)	χ^2^ = 0.712	433 (88.2%)	58 (11.8%)	χ^2^ = 0.402
Age										
	18–35 years	154 (54.0%)	131 (46.0%)	***p*** **= 0.010**	269 (97.8%)	6 (2.2%)	*p* = 0.728	219 (80.5%)	53 (19.5%)	***p*** **< 0.001**
	36–50 years	132 (53.4%)	115 (46.6%)	χ^2^ = 11.352	234 (96.7%)	8 (3.3%)	χ^2^ = 1.306	210 (86.8%)	32 (13.2%)	χ^2^ = 42.543
	51–65 years	156 (52.7%)	140 (47.3%)		277 (96.2%)	11 (3.8%)		257 (92.1%)	22 (7.9%)	
	66+ years	133 (66.5%)	67 (33.5%)		175 (96.7%)	6 (3.3%)		172 (99.4%)	1 (0.6%)	
Circadian preference										
	Definitely morning	65 (59.1%)	45 (40.9%)	***p*** **= 0.002**	103 (97.2%)	3 (2.8%)	***p*** **= 0.001**	101 (99.0%)	1 (1.0%)	***p*** **< 0.001**
	Morning	168 (62.7%)	100 (37.3%)	χ^2^ = 17.039	252 (99.2%)	2 (0.8%)	χ^2^ = 17.715	236 (93.3%)	17 (6.7%)	χ^2^ = 61.127
	Intermediate	167 (58.8%)	117 (41.2%)		265 (97.1%)	8 (2.9%)		245 (91.8%)	22 (8.2%)	
	Evening	112 (49.8%)	113 (50.2%)		214 (96.8%)	7 (3.2%)		186 (85.3%)	32 (14.7%)	
	Definitely evening	58 (44.3%)	73 (55.7%)		114 (91.2%)	11 (8.8%)		87 (70.7%)	36 (29.3%)	

Significant *p*-values in bold.

**Table 5:** Logistic regression analyses with chronic sleep problems (duration ≥ 3 months), short workday sleep duration (<6 h) and long social jetlag (≥2 h) as dependent variables, and sex, age, education, and circadian preference as independent variables.

		Chronic Sleep Problems		Short Workday Sleep Duration		Long Social Jetlag	
		Crude	Adjusted	Crude	Adjusted	Crude	Adjusted
		**OR (95% CI)**	**OR (95% CI)**	**OR (95% CI)**	**OR (95% CI)**	**OR (95% CI)**	**OR (95% CI)**
Sex							
	Female	**1.68 (1.31–2.16)**	**1.80 (1.39–2.33)**	1.32 (0.64–2.73)	1.54 (0.73–3.24)	0.88 (0.59–1.31)	0.83 (0.54–1.28)
	Male	1	1	1	1	1	1
Age							
	18–35 years	1	1	1	1	1	1
	36–50 years	1.03 (0.73–1.45)	1.16 (0.82–1.65)	1.48 (0.50–4.43)	1.56 (0.51–4.76)	0.63 (0.39–1.01)	**0.59 (0.35–0.97)**
	51–65 years	1.06 (0.76–1.46)	1.24 (0.88–1.74)	1.78 (0.64–4.94)	2.22 (0.78–6.32)	**0.36 (0.21–0.61)**	**0.40 (0.22–0.67)**
	66+ years	**0.60 (0.41–0.87)**	0.75 (0.51–1.11)	1.67 (0.53–5.21)	2.54 (0.77–8.38)	**0.03 (0.00–0.18)**	**0.03 (0.01–0.22)**
Education							
	Primary school	1	1	1	1	1	1
	Secondary school	1.13 (0.61–2.10)	1.14 (0.61–2.16)	0.61 (0.15–2.46)	0.64 (0.15–2.68)	1.07 (0.41–2.78)	1.04 (0.37–2.98)
	College/university	0.89 (0.49–1.62)	0.82 (0.44–1.53)	0.42 (0.11–1.68)	0.44 (0.11–1.82)	0.71 (0.28–1.84)	0.60 (0.21–1.69)
Circadian preference							
	Definitely morning	0.97 (0.62–1.52)	0.87 (0.55–1.38)	0.91 (0.24–3.53)	0.81 (0.21–3.20)	**0.17 (0.03–0.89)**	**0.17 (0.03–0.89)**
	Morning	0.85 (0.60–1.20)	0.80 (0.56–1.14)	0.23 (0.04–1.15)	0.22 (0.04–1.12)	0.79 (0.41–1.53)	0.83 (0.42–1.63)
	Intermediate	1	1	1	1	1	1
	Evening	**1.45 (1.02–2.06)**	1.41 (0.98–2.02)	0.98 (0.35–2.78)	1.05 (0.37–3.01)	**1.96 (1.10–3.48)**	1.66 (0.92–3.00)
	Definitely evening	**1.78 (1.17–2.70)**	**1.68 (1.10–2.58)**	**3.07 (1.21–7.78)**	**3.56 (1.35–9.38)**	**4.65 (2.59–8.36)**	**3.94 (2.15–7.23)**

In the crude analyses, each independent variable was entered separately, in the adjusted analyses the independent variables were entered together. OR = odds ratio, 95% CI = 95% confidence interval. Significant values in bold.

## Data Availability

Data can be made available upon request.
